# Crystal structure of bis­(1-benzyl-1*H*-1,2,4-triazole) perchloric acid monosolvate

**DOI:** 10.1107/S1600536814024829

**Published:** 2014-11-26

**Authors:** Yong-Qi Qin, Jin-hui Xue, Yuan-Biao Qiao, Zi-Feng Zhang

**Affiliations:** a Laboratory of Medicinal Chemistry, Lvliang University, Lvliang, Shanxi 033001, People’s Republic of China; b Department of Chemistry and Chemical Engineering, Lvliang University, Lvliang, Shanxi 033001, People’s Republic of China

**Keywords:** crystal structure, 1*H*-1,2,4-triazole, perchloric acid, anti­viral activity

## Abstract

The title compound, 2C_9_H_9_N_3_·HClO_4_, was prepared by reaction of 1-benzyl-1*H*-1,2,4-triazole and HClO_4_ in ethanol at room temperature. The asymmetric unit consists of two mol­ecules of 1-benzyl-1*H*-1,2,4-triazole and one of HClO_4_ mol­ecule. The benzene and triazole rings make dihedral angles of 85.45 (8) and 84.76 (8)° in the two mol­ecules. The H-atom position of the perchloric acid mol­ecule is split over two O atoms (real peaks on difference map), with site-occupation factors of 0.5. These H atoms form two classical hydrogen bonds [2.546 (5) and 2.620 (4) Å] with the same N atoms in both mol­ecules. Five inter­molecular non-classical C—H⋯O inter­actions, with C⋯O distances in the range 3.147 (5)–3.483 (5) Å, are found in the crystal structure.

## Related literature   

For the anti­viral activity of triazole derivatives, see: Madan & Taneja (1991 ▶); Borisova *et al.* (2007 ▶) and of polyligand complexes with metals, see: Xu *et al.* (2004 ▶). For a related structure, see: Ji *et al.* (2002 ▶).
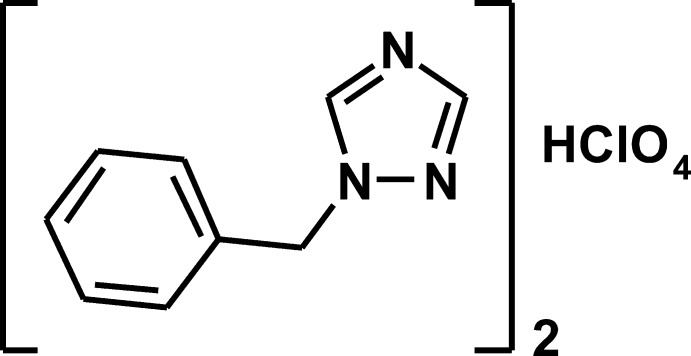


## Experimental   

### Crystal data  

2C_9_H_9_N_3_·HClO_4_
*M* *_r_* = 418.84
Monoclinic, 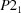

*a* = 5.7173 (10) Å
*b* = 7.7671 (12) Å
*c* = 21.955 (4) Å
β = 95.136 (4)°
*V* = 971.0 (3) Å^3^
*Z* = 2
Mo *K*α radiation
μ = 0.24 mm^−1^
*T* = 296 K
0.25 × 0.23 × 0.20 mm

### Data collection  

Bruker SMART CCD diffractometer
9373 measured reflections
3981 independent reflections
2680 reflections with *I* > 2σ(*I*)
*R*_int_ = 0.050

### Refinement  

*R*[*F* ^2^ > 2σ(*F* ^2^)] = 0.053
*wR*(*F* ^2^) = 0.137
*S* = 1.05
3981 reflections
263 parameters
1 restraint
H-atom parameters constrained
Δρ_max_ = 0.35 e Å^−3^
Δρ_min_ = −0.39 e Å^−3^
Absolute structure: Flack (1983 ▶), 873 Friedel pairs
Absolute structure parameter: 0.27 (9)

### 

Data collection: *SMART* (Bruker, 1997 ▶); cell refinement: *SAINT* (Bruker, 1997 ▶); data reduction: *SAINT*; program(s) used to solve structure: *SHELXS97* (Sheldrick, 2008 ▶); program(s) used to refine structure: *SHELXL97* (Sheldrick, 2008 ▶); molecular graphics: *SHELXTL* (Sheldrick, 2008 ▶); software used to prepare material for publication: *SHELXTL*.

## Supplementary Material

Crystal structure: contains datablock(s) I. DOI: 10.1107/S1600536814024829/rk2425sup1.cif

Structure factors: contains datablock(s) I. DOI: 10.1107/S1600536814024829/rk2425Isup2.hkl

Supporting information file. DOI: 10.1107/S1600536814024829/rk2425Isup3.cml

**Fig. 1.**
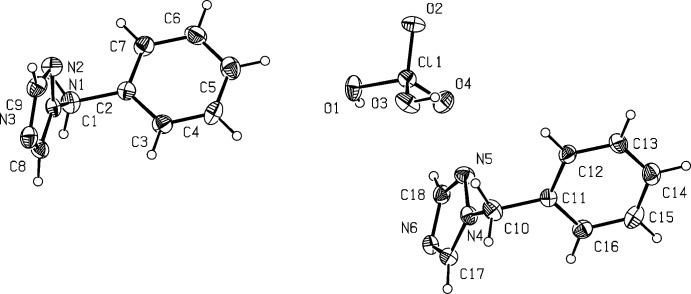
The molecular structure of the title compound with the atom numbering scheme. The displacement ellipsoids are drawn at the 40% probability level H atoms are presented as a small spheres of arbitrary radius.

**Fig. 2.**
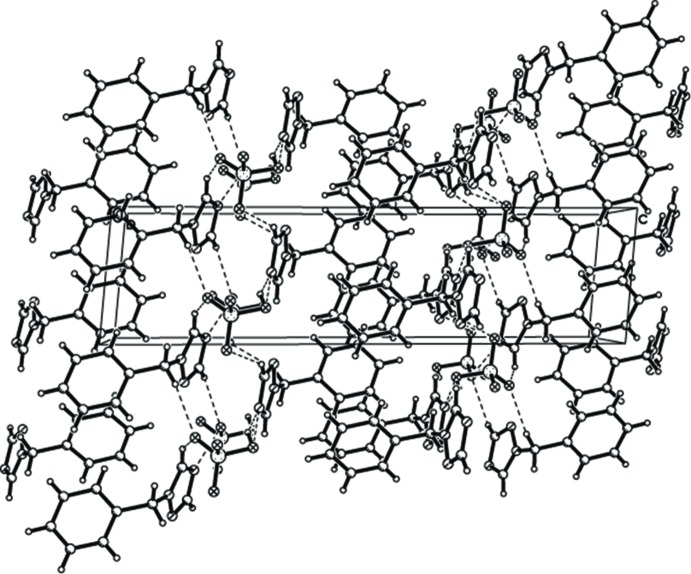
The packing of the title compound, viewed down the b axis, showing short contact (dashed lines).

. DOI: 10.1107/S1600536814024829/rk2425fig1.tif
The mol­ecular structure of the title compound with the atom numbering scheme. The displacement ellipsoids are drawn at the 40% probability level H atoms are presented as a small spheres of arbitrary radius.

b . DOI: 10.1107/S1600536814024829/rk2425fig2.tif
The packing of the title compound, viewed down the *b* axis, showing short contact (dashed lines).

CCDC reference: 1033730

Additional supporting information:  crystallographic information; 3D view; checkCIF report

## Figures and Tables

**Table 1:** Hydrogen-bond geometry (, )

*D*H*A*	*D*H	H*A*	*D* *A*	*D*H*A*
O3H3*A*N6^i^	0.82	2.04	2.620(4)	127
O1H1N3^ii^	0.82	1.78	2.546(5)	154
C10H10*B*O4	0.97	2.35	3.282(5)	160
C9H9O2^iii^	0.93	2.52	3.352(6)	150
C1H1*A*O2^iv^	0.97	2.56	3.483(5)	158
C17H17O4^v^	0.93	2.43	3.147(5)	134
C18H18O3^vi^	0.93	2.44	3.185(5)	137

Symmetry codes: (i) 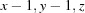; (ii) 
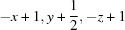
; (iii) 
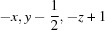
; (iv) 
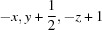
; (v) 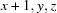; (vi) 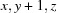.

## supplementary crystallographic information

### S1. Comment

It is well known, that triazole derivatives possess anti-viral activities (Madan & Taneja, 1991; Borisova *et al.*, 2007). A materials about anti-viral activities of the such polyligand complexes with metals were published Xu *et al.*, 2004. These facts were of a reason for structural study of 1-benzyl-1*H*-1,2,4-triazole HClO_4_ solvate.

In the molecule of the title compound (Fig. 1), bond lengths and angles are generally normal in benzene and 1,2,4-triazole ring (Ji *et al.*, 2002). The torsion angles of C2—C1—N1—C8 and N1—C1—C2—C3 are -108.2 (4)° and 60.3 (5)°, respectively; C11—C10—N4—C17 and N4—C10—C11—C12 are 115.2 (4)° and 124.3 (4)° respectively. The dihedral angle formed by the benzene and the triazole ring is 85.45 (8)° and 84.76 (8)° for first and second molecules respectively.

There are some classical O—H···N and non-classical C—H···O intermolecular interactions stabilize the title crystal structure (Fig. 2).

### S2. Experimental

The title compound was prepared by reaction of benzyl chloride (0.05 mol), triazole (0.05 mol), potassium carbonate (0.06 mol) and potassium iodide (0.5 g) in the acetone solution (40 ml) at 333 K for 8 h, filtering, evaporating the solvent, affording the tile compound (6.2 g, yield 78%) by solidification in the etanol solution of HClO_4_ (5%, 15 ml). In the reaction, potassium carbonate can powerfully adsorb HCl to promote the reaction and potassium iodide is catalyst. Single crystals of the title compound suitable for X-ray measurements was obtained by recrystallization from ethanol/acetone (*v*/*v* = 1:1) at room temperature.

### S3. Refinement

The H atoms were fixed geometrically and allowed to ride on their attached atoms, with C—H distances = 0.93–0.97 Å; O—H = 0.82 Å and *U*_iso_(H) = 1.2–1.5*U*_eq_(C, O).

### Crystal data

**Table:**

2C_9_H_9_N_3_·HClO_4_	*F*(000) = 436
*M**_r_* = 418.84	*D*_x_ = 1.433 Mg m^−^^3^
Monoclinic, *P*2_1_	Mo *K*α radiation, λ = 0.71073 Å
*a* = 5.7173 (10) Å	Cell parameters from 637 reflections
*b* = 7.7671 (12) Å	θ = 3.2–19.6°
*c* = 21.955 (4) Å	µ = 0.24 mm^−^^1^
β = 95.136 (4)°	*T* = 296 K
*V* = 971.0 (3) Å^3^	Rectangle, colourless
*Z* = 2	0.25 × 0.23 × 0.20 mm

### Data collection

**Table:**

Bruker SMART CCD diffractometer	2680 reflections with *I* > 2σ(*I*)
Radiation source: fine-focus sealed tube	*R*_int_ = 0.050
Graphite monochromator	θ_max_ = 28.1°, θ_min_ = 2.8°
φ and ω scans	*h* = −7→7
9373 measured reflections	*k* = −9→9
3981 independent reflections	*l* = −28→28

### Refinement

**Table:**

Refinement on *F*^2^	Hydrogen site location: mixed
Least-squares matrix: full	H-atom parameters constrained
*R*[*F*^2^ > 2σ(*F*^2^)] = 0.053	*w* = 1/[σ^2^(*F*_o_^2^) + (0.0604*P*)^2^ + 0.021*P*] where *P* = (*F*_o_^2^ + 2*F*_c_^2^)/3
*wR*(*F*^2^) = 0.137	(Δ/σ)_max_ < 0.001
*S* = 1.05	Δρ_max_ = 0.35 e Å^−^^3^
3981 reflections	Δρ_min_ = −0.39 e Å^−^^3^
263 parameters	Extinction correction: *SHELXL97* (Sheldrick, 2008), Fc^*^=kFc[1+0.001xFc^2^λ^3^/sin(2θ)]^-1/4^
1 restraint	Extinction coefficient: 0.007 (2)
Primary atom site location: structure-invariant direct methods	Absolute structure: Flack (1983) parameter determined using 873 quotients
Secondary atom site location: difference Fourier map	Absolute structure parameter: 0.27 (9)

### Special details

**Table:**

Geometry. All s.u.'s (except the s.u. in the dihedral angle between two l.s. planes) are estimated using the full covariance matrix. The cell s.u.'s are taken into account individually in the estimation of s.u.'s in distances, angles and torsion angles; correlations between s.u.'s in cell parameters are only used when they are defined by crystal symmetry. An approximate (isotropic) treatment of cell s.u.'s is used for estimating s.u.'s involving l.s. planes.

### Fractional atomic coordinates and isotropic or equivalent isotropic displacement parameters (Å^2^)

**Table:**

	*x*	*y*	*z*	*U*_iso_*/*U*_eq_	Occ. (<1)
Cl1	0.22466 (15)	0.25879 (13)	0.75737 (4)	0.0376 (3)	
O2	−0.0268 (4)	0.2501 (5)	0.75956 (13)	0.0501 (7)	
O3	0.3257 (5)	0.0851 (3)	0.75971 (14)	0.0506 (8)	
H3A	0.2975	0.0380	0.7917	0.076*	0.50
O1	0.2712 (5)	0.3307 (4)	0.69478 (12)	0.0520 (8)	
H1	0.3440	0.4193	0.7039	0.078*	0.50
O4	0.3397 (5)	0.3708 (4)	0.80363 (14)	0.0579 (9)	
N2	0.1586 (5)	0.2466 (5)	0.33197 (15)	0.0446 (8)	
C5	0.1887 (8)	0.5483 (7)	0.5465 (2)	0.0544 (12)	
H5	0.1552	0.5518	0.5871	0.065*	
N3	0.4745 (6)	0.0982 (5)	0.31116 (16)	0.0459 (9)	
C9	0.2415 (8)	0.0996 (6)	0.3156 (2)	0.0474 (11)	
H9	0.1473	0.0032	0.3076	0.057*	
N1	0.3519 (6)	0.3489 (4)	0.33810 (14)	0.0351 (8)	
C1	0.3406 (8)	0.5273 (5)	0.35882 (18)	0.0443 (11)	
H1A	0.2189	0.5877	0.3336	0.053*	
H1B	0.4891	0.5838	0.3539	0.053*	
C6	0.0396 (8)	0.6210 (7)	0.5020 (2)	0.0549 (13)	
H6	−0.0967	0.6744	0.5125	0.066*	
C2	0.2893 (7)	0.5377 (5)	0.42460 (18)	0.0364 (9)	
C8	0.5381 (6)	0.2598 (7)	0.32578 (17)	0.0417 (9)	
H8	0.6903	0.3026	0.3271	0.050*	
C4	0.3899 (8)	0.4693 (6)	0.53045 (19)	0.0507 (12)	
H4	0.4927	0.4191	0.5605	0.061*	
C7	0.0878 (7)	0.6168 (6)	0.4414 (2)	0.0460 (11)	
H7	−0.0161	0.6675	0.4117	0.055*	
C3	0.4401 (7)	0.4640 (6)	0.4706 (2)	0.0454 (11)	
H3	0.5770	0.4104	0.4606	0.054*	
N5	0.7031 (5)	0.7708 (5)	0.83783 (15)	0.0417 (8)	
N4	0.8811 (6)	0.6541 (4)	0.84658 (14)	0.0366 (8)	
C18	0.7984 (7)	0.9014 (6)	0.81290 (17)	0.0395 (10)	
H18	0.7188	1.0027	0.8019	0.047*	
C16	0.9431 (7)	0.5756 (6)	0.98327 (19)	0.0420 (10)	
H16	1.0821	0.6247	0.9726	0.050*	
N6	1.0286 (6)	0.8733 (4)	0.80462 (14)	0.0356 (8)	
C11	0.7924 (6)	0.4980 (5)	0.93922 (17)	0.0317 (9)	
C12	0.5874 (7)	0.4219 (6)	0.95742 (18)	0.0405 (10)	
H12	0.4834	0.3681	0.9284	0.049*	
C10	0.8470 (8)	0.4849 (5)	0.87376 (19)	0.0441 (11)	
H10A	0.9882	0.4167	0.8716	0.053*	
H10B	0.7192	0.4259	0.8503	0.053*	
C14	0.6861 (8)	0.5066 (6)	1.0598 (2)	0.0485 (12)	
H14	0.6493	0.5113	1.1002	0.058*	
C17	1.0749 (7)	0.7164 (5)	0.82694 (18)	0.0357 (10)	
H17	1.2192	0.6605	0.8284	0.043*	
C15	0.8882 (8)	0.5809 (6)	1.0437 (2)	0.0507 (12)	
H15	0.9894	0.6352	1.0732	0.061*	
C13	0.5389 (8)	0.4258 (6)	1.01696 (19)	0.0482 (12)	
H13	0.4038	0.3727	1.0284	0.058*	

### Atomic displacement parameters (Å^2^)

**Table:**

	*U*^11^	*U*^22^	*U*^33^	*U*^12^	*U*^13^	*U*^23^
Cl1	0.0384 (5)	0.0346 (5)	0.0404 (5)	0.0025 (5)	0.0060 (4)	0.0025 (5)
O2	0.0300 (13)	0.0547 (19)	0.0666 (19)	0.0054 (16)	0.0088 (12)	−0.0001 (19)
O3	0.0517 (17)	0.0285 (16)	0.075 (2)	0.0139 (14)	0.0271 (15)	0.0174 (15)
O1	0.078 (2)	0.0441 (18)	0.0338 (16)	−0.0108 (16)	0.0065 (15)	0.0126 (13)
O4	0.0575 (19)	0.065 (2)	0.0497 (19)	−0.0129 (17)	−0.0052 (15)	−0.0157 (17)
N2	0.0429 (17)	0.039 (2)	0.052 (2)	0.000 (2)	0.0037 (15)	−0.003 (2)
C5	0.063 (3)	0.053 (3)	0.048 (3)	−0.005 (3)	0.010 (2)	−0.010 (2)
N3	0.057 (2)	0.045 (2)	0.035 (2)	−0.0044 (19)	0.0028 (17)	−0.0077 (17)
C9	0.050 (3)	0.044 (3)	0.047 (3)	−0.010 (2)	−0.005 (2)	−0.008 (2)
N1	0.0440 (19)	0.033 (2)	0.0289 (17)	0.0003 (16)	0.0082 (15)	−0.0037 (15)
C1	0.057 (3)	0.031 (3)	0.045 (3)	−0.001 (2)	0.007 (2)	0.007 (2)
C6	0.042 (3)	0.066 (4)	0.059 (3)	0.001 (2)	0.014 (2)	−0.014 (3)
C2	0.040 (2)	0.029 (2)	0.040 (2)	−0.0019 (18)	0.0032 (18)	−0.0044 (19)
C8	0.041 (2)	0.045 (3)	0.040 (2)	−0.009 (3)	0.0093 (17)	−0.002 (3)
C4	0.065 (3)	0.046 (3)	0.039 (3)	0.003 (2)	−0.011 (2)	−0.003 (2)
C7	0.048 (3)	0.041 (3)	0.048 (3)	0.008 (2)	0.001 (2)	−0.001 (2)
C3	0.043 (2)	0.043 (3)	0.050 (3)	0.009 (2)	0.004 (2)	−0.010 (2)
N5	0.0370 (16)	0.040 (2)	0.049 (2)	0.0052 (18)	0.0065 (14)	0.003 (2)
N4	0.042 (2)	0.033 (2)	0.0354 (18)	−0.0029 (15)	0.0076 (16)	0.0000 (16)
C18	0.044 (2)	0.040 (3)	0.034 (2)	0.007 (2)	0.0028 (18)	0.005 (2)
C16	0.040 (2)	0.039 (2)	0.047 (3)	−0.0060 (19)	0.0015 (19)	0.000 (2)
N6	0.0432 (19)	0.033 (2)	0.0307 (18)	0.0002 (16)	0.0060 (15)	−0.0007 (16)
C11	0.039 (2)	0.022 (2)	0.034 (2)	0.0028 (17)	0.0034 (17)	0.0023 (17)
C12	0.040 (2)	0.045 (3)	0.036 (2)	−0.0062 (19)	0.0009 (18)	0.007 (2)
C10	0.053 (3)	0.033 (2)	0.049 (3)	−0.002 (2)	0.016 (2)	0.008 (2)
C14	0.060 (3)	0.047 (3)	0.040 (2)	0.014 (2)	0.008 (2)	0.014 (2)
C17	0.037 (2)	0.031 (3)	0.039 (2)	−0.0017 (18)	0.0049 (17)	0.0010 (17)
C15	0.062 (3)	0.043 (3)	0.044 (3)	−0.001 (2)	−0.012 (2)	0.002 (2)
C13	0.046 (3)	0.058 (3)	0.043 (3)	−0.005 (2)	0.012 (2)	0.013 (2)

### Geometric parameters (Å, º)

**Table:**

Cl1—O2	1.444 (2)	C4—H4	0.9300
Cl1—O4	1.449 (3)	C7—H7	0.9300
Cl1—O3	1.466 (3)	C3—H3	0.9300
Cl1—O1	1.529 (3)	N5—C18	1.295 (5)
O3—H3A	0.8200	N5—N4	1.363 (4)
O1—H1	0.8200	N4—C17	1.317 (5)
N2—C9	1.299 (6)	N4—C10	1.464 (5)
N2—N1	1.358 (4)	C18—N6	1.362 (5)
C5—C6	1.359 (7)	C18—H18	0.9300
C5—C4	1.377 (6)	C16—C11	1.375 (5)
C5—H5	0.9300	C16—C15	1.390 (6)
N3—C8	1.338 (6)	C16—H16	0.9300
N3—C9	1.345 (5)	N6—C17	1.331 (5)
C9—H9	0.9300	C11—C12	1.402 (5)
N1—C8	1.318 (5)	C11—C10	1.501 (5)
N1—C1	1.462 (5)	C12—C13	1.361 (5)
C1—C2	1.501 (5)	C12—H12	0.9300
C1—H1A	0.9700	C10—H10A	0.9700
C1—H1B	0.9700	C10—H10B	0.9700
C6—C7	1.384 (6)	C14—C13	1.359 (6)
C6—H6	0.9300	C14—C15	1.367 (6)
C2—C7	1.384 (5)	C14—H14	0.9300
C2—C3	1.391 (6)	C17—H17	0.9300
C8—H8	0.9300	C15—H15	0.9300
C4—C3	1.370 (6)	C13—H13	0.9300
			
O2—Cl1—O4	113.29 (19)	C2—C7—H7	119.7
O2—Cl1—O3	110.2 (2)	C4—C3—C2	121.0 (4)
O4—Cl1—O3	112.0 (2)	C4—C3—H3	119.5
O2—Cl1—O1	107.50 (18)	C2—C3—H3	119.5
O4—Cl1—O1	107.82 (19)	C18—N5—N4	104.1 (3)
O3—Cl1—O1	105.54 (17)	C17—N4—N5	110.4 (3)
Cl1—O3—H3A	109.5	C17—N4—C10	127.6 (3)
Cl1—O1—H1	102.2	N5—N4—C10	122.0 (3)
C9—N2—N1	103.2 (3)	N5—C18—N6	112.3 (4)
C6—C5—C4	119.1 (4)	N5—C18—H18	123.9
C6—C5—H5	120.4	N6—C18—H18	123.9
C4—C5—H5	120.4	C11—C16—C15	120.2 (4)
C8—N3—C9	103.0 (4)	C11—C16—H16	119.9
N2—C9—N3	114.6 (4)	C15—C16—H16	119.9
N2—C9—H9	122.7	C17—N6—C18	105.1 (4)
N3—C9—H9	122.7	C16—C11—C12	118.1 (4)
C8—N1—N2	109.7 (3)	C16—C11—C10	122.2 (4)
C8—N1—C1	128.4 (4)	C12—C11—C10	119.7 (4)
N2—N1—C1	121.8 (3)	C13—C12—C11	120.8 (4)
N1—C1—C2	111.6 (3)	C13—C12—H12	119.6
N1—C1—H1A	109.3	C11—C12—H12	119.6
C2—C1—H1A	109.3	N4—C10—C11	112.1 (3)
N1—C1—H1B	109.3	N4—C10—H10A	109.2
C2—C1—H1B	109.3	C11—C10—H10A	109.2
H1A—C1—H1B	108.0	N4—C10—H10B	109.2
C5—C6—C7	120.9 (4)	C11—C10—H10B	109.2
C5—C6—H6	119.5	H10A—C10—H10B	107.9
C7—C6—H6	119.5	C13—C14—C15	120.1 (4)
C7—C2—C3	117.7 (4)	C13—C14—H14	120.0
C7—C2—C1	121.3 (4)	C15—C14—H14	120.0
C3—C2—C1	121.0 (4)	N4—C17—N6	108.1 (3)
N1—C8—N3	109.6 (3)	N4—C17—H17	125.9
N1—C8—H8	125.2	N6—C17—H17	125.9
N3—C8—H8	125.2	C14—C15—C16	120.2 (4)
C3—C4—C5	120.6 (4)	C14—C15—H15	119.9
C3—C4—H4	119.7	C16—C15—H15	119.9
C5—C4—H4	119.7	C14—C13—C12	120.6 (4)
C6—C7—C2	120.6 (4)	C14—C13—H13	119.7
C6—C7—H7	119.7	C12—C13—H13	119.7
			
N1—N2—C9—N3	0.6 (5)	C18—N5—N4—C17	−0.1 (4)
C8—N3—C9—N2	−0.5 (5)	C18—N5—N4—C10	−179.5 (3)
C9—N2—N1—C8	−0.4 (4)	N4—N5—C18—N6	0.7 (4)
C9—N2—N1—C1	−177.4 (4)	N5—C18—N6—C17	−1.0 (4)
C8—N1—C1—C2	−108.2 (4)	C15—C16—C11—C12	−1.5 (6)
N2—N1—C1—C2	68.1 (5)	C15—C16—C11—C10	−178.3 (4)
C4—C5—C6—C7	−0.1 (8)	C16—C11—C12—C13	0.4 (6)
N1—C1—C2—C7	−118.1 (4)	C10—C11—C12—C13	177.2 (4)
N1—C1—C2—C3	60.3 (5)	C17—N4—C10—C11	115.2 (4)
N2—N1—C8—N3	0.2 (4)	N5—N4—C10—C11	−65.5 (5)
C1—N1—C8—N3	176.9 (4)	C16—C11—C10—N4	−58.9 (5)
C9—N3—C8—N1	0.2 (5)	C12—C11—C10—N4	124.3 (4)
C6—C5—C4—C3	0.1 (8)	N5—N4—C17—N6	−0.5 (4)
C5—C6—C7—C2	0.2 (7)	C10—N4—C17—N6	178.9 (4)
C3—C2—C7—C6	−0.2 (6)	C18—N6—C17—N4	0.8 (4)
C1—C2—C7—C6	178.2 (4)	C13—C14—C15—C16	0.6 (7)
C5—C4—C3—C2	−0.2 (7)	C11—C16—C15—C14	1.0 (6)
C7—C2—C3—C4	0.2 (6)	C15—C14—C13—C12	−1.8 (7)
C1—C2—C3—C4	−178.2 (4)	C11—C12—C13—C14	1.3 (6)

### Hydrogen-bond geometry (Å, º)

**Table:**

*D*—H···*A*	*D*—H	H···*A*	*D*···*A*	*D*—H···*A*
O3—H3*A*···N6^i^	0.82	2.04	2.620 (4)	127
O1—H1···N3^ii^	0.82	1.78	2.546 (5)	154
C10—H10*B*···O4	0.97	2.35	3.282 (5)	160
C9—H9···O2^iii^	0.93	2.52	3.352 (6)	150
C1—H1*A*···O2^iv^	0.97	2.56	3.483 (5)	158
C17—H17···O4^v^	0.93	2.43	3.147 (5)	134
C18—H18···O3^vi^	0.93	2.44	3.185 (5)	137

Symmetry codes: (i) *x*−1, *y*−1, *z*; (ii) −*x*+1, *y*+1/2, −*z*+1; (iii) −*x*, *y*−1/2, −*z*+1; (iv) −*x*, *y*+1/2, −*z*+1; (v) *x*+1, *y*, *z*; (vi) *x*, *y*+1, *z*.
