# Supplementary figures and images for: Crystal structure of bis­(1-benzyl-1H-1,2,4-triazole) perchloric acid monosolvate
Source: Acta Crystallogr Sect E Struct Rep Online. 2014 Nov 26;70(Pt 12):o1290–1. doi: 10.1107/S1600536814024829 (PMC4257456; doi:10.1107/S1600536814024829)

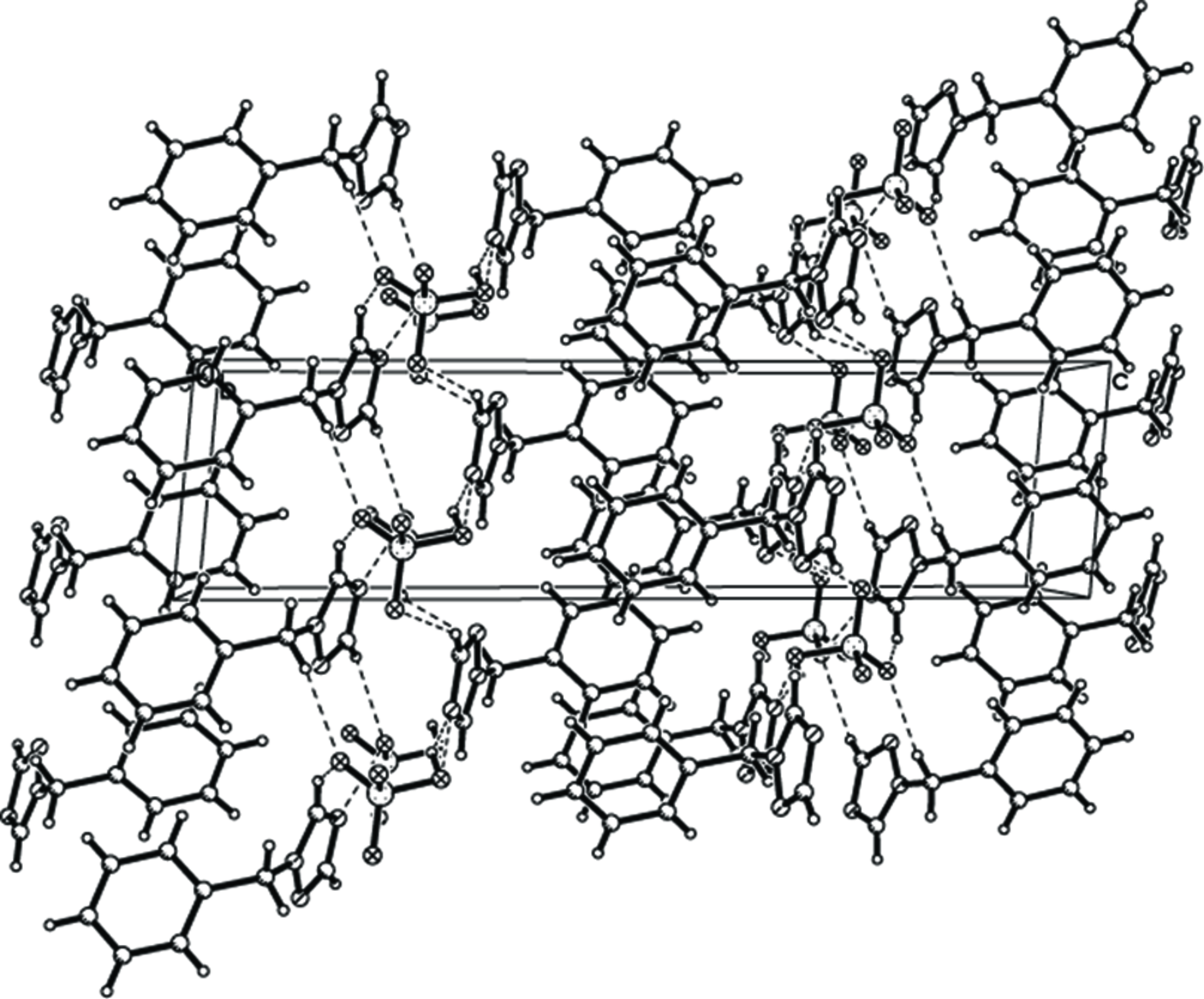

Supplement: Supplementary file 5 [file e-70-o1290-fig2.tif]
